# MBL2 gene polymorphism rs1800450 and rheumatic fever with and without rheumatic heart disease: an Egyptian pilot study

**DOI:** 10.1186/s12969-018-0245-x

**Published:** 2018-04-13

**Authors:** Maher Hassan Gomaa, Shawkey Sadik Ali, Aya Mohamed Fattouh, Hala Salah Hamza, Mohamed Mohamed Badr

**Affiliations:** 1 Biochemistry Department-Faculty of Pharmacy (Boys), Al-Azhar University, Almokhayam Aldaem Street, 6th Province - 13465 Nasr City, Cairo, Egypt; 2 Department of Pediatrics, Kasr Al-Aini School of Medicine, Cairo University, P.O. Box 99, Manial El-Roda, Cairo, 11553 Egypt

**Keywords:** Rheumatic fever, Rheumatic heart disease, MBL2 gene polymorphism

## Abstract

**Background:**

Rheumatic fever (RF) is the result of an autoimmune response to pharyngitis caused by infection with *Streptococcus pyogenes*. RF is most prevalent in Africa and the Middle East. Rheumatic heart disease (RHD) is the most serious complication of RF. Mannose-binding lectin 2 gene (MBL2) has been reported to be correlated with different cardiac conditions. In Egyptian patients as a new studied ethnic population, it is the first time to evaluate the association between MBL2 gene polymorphism rs1800450 and RF with and without RHD.

**Methods:**

One hundred and sixty RF patients (80 with RHD and 80 without RHD) and eighty healthy ethnically matched controls were studied. MBL2 (rs1800450) was genotyped by real-time PCR using TaqMan® allele discrimination assay. The MBL level was measured by ELISA. Westergren erythrocytes sedimentation rate (ESR), anti-streptolysin O titer (ASOT), C-reactive protein (CRP) and complements (C3 and C4) were determined.

**Results:**

The AA genotype with high production of MBL was associated with increased risk of RHD more than the B allele carrying subjects. However, MBL2 genotype related to the low production of MBL was more frequently observed in those patients without RHD.

**Conclusions:**

Our results suggested the involvement of MBL2 (rs1800450) polymorphism and its protein in RHD pathogenesis. Also, it might be a promising future strategy to utilize this polymorphism to help differentiate patients with RHD from those without RHD.

## Background

Globally, rheumatic heart disease (RHD) is still the leading cause of acquired heart disease among the young [1]. Many echocardiographic screening studies put the prevalence of RHD at 8–57 out of 1000 children meaning that the prevalence may rest closer to 62–78 million individuals worldwide with up to 1.4 million deaths each year [2, 3]. A large African study indicated that RHD prevails as the most frequent cause of heart failure (HF) among children and young adults [4].

Despite being preventable and imminently treatable disease, sadly, RHD can go undetected until patients’ presentation with debilitating HF. At this stage, surgery is the only possible treatment option [5]. Patients living in poor countries have limited or no access to expensive heart surgery. To prevent these serious complications, it is very important to find out the most patients vulnerable to RHD to receive their further medical follow-up.

The main hypothesis for RF development is an autoimmune reaction resulting from molecular mimicry between streptococcal antigen and cardiac, articular and central nervous system proteins [6].

The innate immune response provides the first line of defense against *Streptococcus pyogenes* infections, in the case of RF, with complement cascade activation. Mannose-binding lectin (MBL) is an acute phase inflammatory protein and functions as a soluble pathogen recognition receptor. It binds to a wide variety of sugars on the surface of pathogens and plays a major role in innate immunity due to its ability to opsonize pathogens, enhancing their phagocytosis and activating the complement cascade via the lectin pathway [7]. N-acetylglucosamine, one of the main constituents of the streptococci cell wall [8] and of human heart valves [9] is a strong ligand for MBL.

Upon binding of the MBL’s higher-order oligomers to the identified carbohydrates moieties, they form complexes with MBL-associated serine proteases (MASPs), which help in activation of the complement system through the lectin pathway and generation of multiple opsonic and inflammatory fragments, which ultimately result in phagocytosis and several immune-mediated reactions [7].

The MBL2 gene is located on the chromosome 10 and comprises four exons. Inherited MBL insufficiency, which results in impaired innate immune function and enhanced susceptibility to infection, is essentially caused by three structural variants in exon 1. These three polymorphisms alter significantly the serum concentrations of MBL, with the variant alleles (D, B, C) resulting from mutations in codons 52, 54 and 57, respectively. The variant alleles are collectively named O, and the normal allele, A. The allele D changes CGT to TGT, changing arginine to cysteine (p.Arg52Cys). The B allele changes GGC to GAC and causes an amino acid replacement of glycine to aspartic acid (p.Gly54Asp) while the allele C changes GGA to GAA substituting glycine to glutamic acid (p.Gly57Glu) [7, 10].

Single nucleotide polymorphism (SNP) B, as it designates an exchange of the glycine in the glycine-rich motif, is associated with a decreased stability of the collagenous region of the protein. Although this MBL2 variant is indeed able to form multimers [11], they are more rapidly degraded and exist primarily as lower order oligomers. Functionally, they have a lower binding capacity to N-acetylglucosamine and do not activate complement [12], presumably due to lacking the capability of binding to MASPs [13].

The information surrounding the role of MBL2 polymorphism in RF pathogenesis remains insufficient. Few previous studies on limited ethnic population showed a direct variation about the MBL2 genotypes associated with the risk of RF and RHD [10]. Based on these findings, the current study was the 1st to demonstrate the association between MBL2 gene polymorphism and RF in Egyptians of different ethnic groups than the most of the studies and as a part of North Africa and the Middle East. And to find if there is a clinical value for this SNP to be predictive for RF complications.

## Methods

### Patients and control

This study was conducted on 160 patients with a history of RF chosen from outpatient RF Clinic, Abol-Reich Hospital, Kasr Al-Aini School of Medicine; Cairo University at the period between January 2015 and June 2015. RF patients were divided into 80 patients with RHD and 80 patients without RHD. Our patients were cases diagnosed, 4 to 12 months before enrolled in the study and not acute RF. All included patients were on monthly intramuscular injection of long-acting penicillin (benzathine penicillin) since their first diagnosis. No one required valvular heart surgery as no one was in HF. The control group included eighty healthy subjects matched with the patients for the ethnic, geographic and socioeconomic background. The study was approved by ethical committee of Abol-Reich Hospital. A written informed consent was obtained from all study subjects or their parents. The diagnosis of RF was made using the modified Jones criteria [14]. Patients presenting infective endocarditis, congenital heart diseases or other inflammatory or immunological disorders were excluded from this study. All the patients were subjected to complete history taking, thorough clinical examination as well as revision of their pre-registered data which included their laboratory investigations. Echocardiography was performed in all patients to assess the valve affection and all patients with RHD had a history of mild to moderate mitral valve regurge. The diagnosis of RHD was based on World Heart Federation criteria [15]. Overnight fasting blood samples from control and all patients were obtained. Each blood sample was distributed into three tubes; the first tube contained citrate, used for determination of 1st-hour erythrocytes sedimentation rate (ESR) using Westergren method [16]. The second tube was allowed to clot for 30 min and then centrifuged at 4000 rpm for 10 min. The obtained sera were used for determination of anti-streptolysin O titer (ASOT), C-reactive protein (CRP), complements (C3 and C4) by turbidimetric immunoassay method using commercially available kits (BioSystems S.A., Costa Brava, Barcelona, Spain). Serum MBL concentration was measured by enzyme-linked immunosorbent assay (ELISA) using commercial kits (Boster Biological Technology Co., Ltd., Pleasanton, USA) according to manufacturer’s instructions and recommendations.

### Genetic analysis

The third tube contained EDTA, used for genomic DNA (gDNA) extraction and purification using Gene JET™ Whole Blood DNA Purification Mini Kit (Thermo Fisher Scientific Inc., USA) and then stored at − 80 °C until analyzed. The gDNA concentration and purity (A260/280) for each sample was determined using NANODROP™ 2000 spectrophotometer (Thermo Fisher Scientific Inc., USA). The extracted DNA was genotyped for MBL2 (AB; codon 54, rs1800450) exon 1 polymorphism by StepOne™ (Applied Biosystems, CA, USA) real-time polymerase chain reaction (PCR) using TaqMan® allele discrimination assay (Applied Biosystems, CA, USA). Probes and primers were designed by Applied Biosystems (ID: C_2336609_20), context sequence TGGTTCCCCCTTTTCTCCCTTGGTG[**C/T**]CATCACGCCCATCTTTGCCTGGGAA. Further details are restricted to Applied Biosystems and cannot be provided. TaqMan PCR was performed in a total volume of 20 μl (20 ng of DNA, 10 μl TaqMan master mix and 0.5 μl TaqMan® allele discrimination assay) placed in 48-well plates. Thermal cycling conditions were initiated by AmpliTaq Gold® polymerase activation at 95 °C for 10 min, followed by 45 cycles (denaturation at 95 °C for 15 s, then annealing/extension at 60 °C for 1 min). After PCR amplification, an endpoint plate read was carried out using the Applied Biosystems real-time PCR system. The fluorescence measurements that made during the plate read used by the sequence detection system software to plot the fluorescence values based on the signals from each well. The plotted fluorescence signals indicated which alleles are in each sample.

### Statistical analysis

GraphPad Prism 6.0 (GraphPad software 2010, San Diego, USA) was used for analysis. Data of the groups were analyzed by D’Agostino and Pearson omnibus normality test to determine the normal distribution pattern. Normally distributed variables are presented as mean ± SE. Skewed distributed variables are presented as median (inter-quartile range). All results were compared by Kruskal-Wallis test (with multiple comparisons using Dunn’s test). Correlations between variables were tested using the Spearman’s correlation coefficients. The genotypes distribution for polymorphism was analyzed for deviation from the Hardy–Weinberg equilibrium (HWE) and any deviation between the observed and expected frequencies was tested for significance using the χ^2^ test. Differences in genotype and allele frequencies between groups were analyzed using the Fisher’s exact tests. In addition, we calculated the odds ratios (ORs) and 95% confidence intervals (CIs) regarding the presence RHD with respect to the existence of the polymorphism. All statistical tests were two-sided. A *p*-value < 0.05 was considered to be statistically significant. The receiver operator characteristic (ROC) curve was constructed to obtain the diagnostic accuracy, cutoff values, sensitivity and specificity for MBL. As an accuracy indicator, the area under the curve (AUC) was used for assessment of the diagnostic performance.

## Results

Regarding ASOT, 1st-h ESR, CRP and C3, significantly increased values were obtained in RF patients without RHD as compared to control. Also, RF patients with RHD showed significantly increased values concerning to 1st-h ESR, CRP, C3 and MBL as compared to controls. In addition, MBL showed significantly increased values in RF patients with RHD as compared to both patients without RHD and controls. However, a significant decrease of CRP was obtained in RF patients with RHD as compared to those without RHD as shown in Table 1.

**Table 1:** Characteristics of the 240 study subjects

Characteristics		Controls (*n* = 80)	RF (*n* = 160)	
			Without RHD (*n* = 80)	With RHD (*n* = 80)
Gender	Female n (%)	48 (60.0)	46 (57.5)	48 (60.0)
	Male n (%)	32 (40.0)	34 (42.5)	32 (40.0)
Age (years)		15.2 ± 0.29	14.5 ± 0.43	14.3 ± 0.33
ASOT (IU/ml)		29.0 (11.3–97.0)	116 (26.3–172) ^a^	63.0 (19.0–127)
1st-h ESR (mm/h)		6.00 (5.00–8.00)	21.5 (13.25–31.50) ^a^	16.5 (11.00–25.0) ^a^
CRP (mg/l)		2.90 (1.70–3.87)	6.55 (5.75–7.37) ^a^	4.30 (3.10–6.25) ^a, b^
C3 (mg/dl)		108.0 (98.0–122)	122.5 (111–136) ^a^	127.5 (113–139) ^a^
C4 (mg/dl)		30.5 (21.0–36.8)	31.5 (27.3–37.8)	32.0 (26.0–36.0)
MBL (ng/ml)		450.6 (290.2–563.1)	591.2 (478.0–1005)	1614 (1419–1945) ^a, b^

Data are presented as n (%), mean ± SE or median (inter-quartile range)

^a^ significant from control group, ^b^ significant from RF without RHD group

Using ROC curve analysis, we found that the critical serum level of MBL associated with the risk of RHD was 1383 ng/ml (AUC = 0.74, SE = 0.037, 95% CI = 0.67 to 0.82, p = < 0.0001, 73.3% sensitivity and 80% specificity).

The RF patients showed negative significant correlations between (MBL) against ASOT (r − 0.22, p 0.013), 1st-hr ESR (r − 0.2, p 0.026), and CRP (r − 0.25, p 0.005). However, MBL showed negative insignificant correlations with C3 (r − 0.03, p 0.757) and C4 (r 0.016, p 0.86).

The genotype distribution for the studied SNP (AB; codon 54, rs1800450) showed no deviation from HWE. Higher frequency of the AA genotype was present in patients with RHD when compared to controls. However, a lower frequency of AB and BB genotypes was observed in RHD patients as compared to healthy controls. In addition, a lower frequency of the B allele was observed in RF patients with RHD when compared to the controls. Patients without RHD showed insignificant differences in the frequency of all genotypes and B allele when compared to controls, as illustrated in Tables 2 and 3.

**Table 2:** Distribution of MBL2 genotypes (rs1800450) in patients with history of RF and controls

	Controls (*n* = 80)	RF (*n* = 160)		p	OR	95% CI
		Without RHD (*n* = 80)	With RHD (*n* = 80)			
Genotypes						
AA n (%)	44 (55)	42 (52.5)	69 (86.25)	1.00 ^a^	0.90	0.37–2.17
				0.0003 ^b^	5.13*	2.10–12.5
AB n (%)	24 (30)	34 (42.5)	11 (13.75)	0.50 ^a^	1.48	0.57–3.84
				0.02 ^b^	0.29*	0.11–75.0
BB n (%)	12 (15)	4 (5)	0 (0)	0.26 ^a^	0.29	0.06–1.58
				0.001 ^b^	0.03*	0.002–0.60
HWE	χ^2^ = 3.3, *p* > 0.05	χ^2^ = 0.38, *p* > 0.05	χ^2^ = 0.44, *p* > 0.05			

OR of AA: by comparison between AA versus (AB+BB) (dominant model)

OR of AB: by comparison between AB versus AA (co-dominant model)

OR of BB: by comparison between BB versus (AA+AB) (recessive model)

^a^ RF without RHD versus controls. ^b^ RF with RHD versus controls

* significant difference at *p* < 0.05

**Table 3:** Distribution of MBL2 alleles (rs1800450) in patients with history of RF and controls

	Controls (*n* = 80)	RF (*n* = 160)		p	OR	95% CI
		Without RHD (*n* = 80)	With RHD (*n* = 80)			
Allele						
A n (%)	112 (70)	118 (73.8)	149 (93.1)			
B n (%)	48 (30)	42 (26.2)	11 (6.9)	0.720 ^a^	0.83	0.42–1.65
				0.0001^b^	0.17*	0.08–0.37

a RF without RHD versus controls. ^b^ RF with RHD versus controls

* significant difference at *p* < 0.05

In this study, RF group was classified according to the MBL2 gene (rs1800450) genotypes into subjects carrying AA genotype and subjects carrying AB+BB genotypes. As shown in Table 4, regarding C4, a significant difference was found between AA of RF group as compared to AB+BB. Looking at the MBL level, the genotype AA in RF group was associated with highly significant concentrations of MBL in serum as compared to individuals carrying the B allele (AB+BB) in the same group. In genotype AB+BB individuals, the MBL levels were reduced 4 times than AA genotype.

**Table 4:** Biochemical and demographic characteristics of RF patients according to MBL2 gene (rs1800450) genotypes

		RF patients	
		AA	AB+BB
Age (years)		14.44 ± 0.30	14.35 ± 0.55
ASOT (IU/ml)		64.5 (19–139.5)	113 (28–189)
1st-h ESR (mm/h)		17 (11–25)	23.5 (14–42.25)
CRP (mg/l)		5.1 (3.5–6.8)	6.2 (5.1–7.9)
C3 (mg/dl)		122.5 (112.8–135.0)	134 (110.8–146)
C4 (mg/dl)		32 (26–36)	25 (19.75–30.25) ^a^
MBL (ng/ml)		1544 (1328–1906)	412.9 (338–501.2) ^a^

Data are presented in mean ± SE or median (inter-quartile range)

^a^ significant difference between AB+BB and AA at *p* < 0.05

## Discussion

Despite the vital role of the lectin pathway in complement activation and host defense against infection and autoimmunity, studies on the significance of components of this pathway in RF and RHD are still scarce [17, 18].

In the current study, regarding ASOT, significantly increased values were found in RF group without RHD as compared to control, which was confirmed by Sainani and Sainani, [19] who reported that the significant increase of ASOT in RF group as compared to control may be explained by recent streptococcal infection in RF patients.

In agreement with Kumar [20] and Farghaly et al., [21], concerning the 1st-h ESR and CRP, there were significantly increased values in RF group as compared to control. These elevations may be explained by the ongoing inflammatory nature of the disease as suggested by Gölbasi et al., [22], Veasy and Tani, [23] and Chiu-Braga et al., [24].

Given the level of C3, it was significantly increased in RF group than the control subjects which is in agreement with Schafranski et al., [25, 26] who suggested the presence of continuous inflammation with probable complement activation. The C3 is an acute phase protein which significantly increases in response to infections or to an inflammatory reaction.

However, the involvement of complement system in RF and RHD has been shown in different studies. Antibodies found in RF patients’ sera that show molecular mimicry between streptococcal and human antigens were able to cause human fibroblasts lyses in vitro in the presence of complement [25].

These results confirmed the involvement of complement system components in the pathogenesis of RF as described by Schafranski et al., [26].

As the best to our knowledge, this is the 1st study in Egypt evaluating MBL levels and MBL2 genotype (A/B; codon 54, rs1800450) in the development of RF.

In the current study, the level of MBL was significantly higher in patients with RHD than both controls and patients without RHD, indicating the high MBL levels could be a cause of undesirable complement activation in carditis patients, contributing to the pathogenesis of rheumatic cardiomyopathy [25]. This result may encourage further studies on anti-MBL as an interesting alternative therapy for patients with this disease.

The negative significant correlation between serum MBL and ASOT in this study could be explained by the subsequent triggering of MBL to the complement system that may be responsible for either a direct complement-dependent killing of the bacteria or increased phagocytosis through the opsonization [27]. These findings confirmed the positive impact of MBL in streptococcal innate immune defense.

The current study showed a significant negative correlation between MBL and ESR that may be explained by either the expression of functional MBL protein is largely genetically determined by polymorphisms found in the first exon of the structural gene [28] or by the characteristic pattern of ESR in rheumatic disease [29].

Our results disclosed a negative significant correlation between MBL and CRP levels that may be related to the following two different functional mechanisms: 1) MBL is involved in the lectin pathway while CRP is involved in the classical complement activation pathway. 2) Although, both MBL and CRP have a common terminal pathway, MBL mainly plays a role in the early responses of the host innate immune system, while CRP mainly plays a role in the late stage of adaptive immune responses [30].

However, in the current study, there were insignificant correlations between C3, C4 and MBL levels, indicating that elevated MBL concentration might be an independent event [31].

In the present study, the homozygous wild genotype AA was more prevalent in patients with RHD than healthy controls. In addition, the frequency of variant allele B was significantly lower in patients with RHD than controls. These findings were in accordance with Schafranski et al., [26] and Reason et al., [32], who concluded that the AA genotype could be involved in the development of cardiac manifestation in RF and the relationship was due to genetic effect of MBL2 polymorphisms rather than to an acute phase reaction. They also suggested a role for AA MBL2 genotype in the susceptibility to rheumatic carditis.

Many previous studies reported the association between MBL deficiency and risk of recurrent different infections [33]. While RF is a result of untreated streptococcal pharyngeal infection [6], previous studies [26, 32] and our results suggested an association between high MBL levels and RF that could be explained by the auto immune nature of RF and RHD [34]. Moreover, the role of MBL in the pathogenesis of rheumatic carditis could be related to the stressful conditions and the inflammatory processes of this carditis which could result in glycosylation of the self-cell surfaces [35]. In the heart valves of RF patients, there is a lot of myxomatous tissue which rich in hyaluronic acid. The N-acetylglucosamine is a major constituent of this hyaluronic acid. So, it can act as a ligand for the high levels of MBL that argue the chronic inflammatory activity present in the RHD individuals and contribute to valve injury through complement activation [9]. In addition, MBL may act as an immunomodulatory molecule, inducing a higher secretion of cytokines by macrophages [36].

Thus, the high blood concentrations of MBL could be considered as a double-edged sword molecule in the physiopathology of RF and rheumatic carditis, on one hand conferring protection against the initial infection by rheumatogenic *Streptococci* which is good, but on the other hand eliciting inflammation and complement dependent tissue damage in the chronic stage of the disease [10].

In contrast to the previous results, in an initial study in Chinese patients, there was no association between the B allele and RHD [37]. However, they suggested an assumed role for MBL deficiency in the progression of RHD, by considering their patient’s age of onset of heart disease. The mean age of onset of cardiac symptoms of patients with the deficient B allele was significantly lower compared with patients with MBL2 genotype AA. These findings supported the hypothesis that MBL deficiency caused by the B allele could facilitate the development and accelerate the progression of RHD in younger people. In addition, higher levels of serum MBL in control group as compared to RHD patients were reported by Scalzi et al. [38] in Yemen population.

Also, in another study on patients from São Paulo, Brazil who suffered from chronic severe rheumatic aortic regurgitation (AR), Ramasawmy et al., [39] observed that B allele had similar frequencies in both AR patients and controls. They postulated that continuous exposure to *Streptococcus* antigens in MBL-deficient individuals would lead to the subsequent abnormal immune response against heart proteins, leading to rheumatic AR. Studies done on MBL2 gene polymorphisms in RHD could be summarized in Table 5.

**Table 5:** Studies on MBL2 gene polymorphism in RHD

Polymorphism	Population	Polymorphism association	Refs
− 221 XY A (52C, 54G, 57G), O (52T, 54A, 57A)	Brazilian	RHD patients with mitral valve lesion showed an association with the A allele	[26, 32]
A (52C, 54G, 57G), O (52T, 54A, 57A)	Chinese	RHD displayed no association with the B allele	[37]
A (52C, 54G, 57G), O (52T, 54A, 57A)	Brazilian	RHD patients with AR displayed an association with the O allele	[39]

The contradiction between these results could be attributed to the difference in ethnicity, clinical manifestations of the studied subjects or due to the difference in the sample size.

In the present study, it was found that the AA genotype of RF patients was associated with high levels of MBL as compared to subjects carrying AB+BB in the same group indicating the suppressive effect of the mutant B allele on the levels of serum MBL that was in agreement with Reason et al., [32] and Schafranski et al., [26].

Some limitations should be considered when interpreting this study results. First, the sample size of the selected patients is not large enough to give a bigger picture about the distribution of MBL2 genotypes. So, further larger sample studies are recommended to verify these findings in Egyptians and other Mediterranean ethnics. Second, the selected SNP was based on the scientific literature, other SNPs may be more prominently implicated in our study included ethnics. Third, this study was independent on patients’ follow-up while the long-term follow-up of patients with RF without valve consequence is necessary in order to identify which patients actually progress to RHD and the risk factors associated with it. Fourth, the age of our studied population may not allow a homogeneous representation of the population with RF.

## Conclusions

The AA genotype associated with a higher production of MBL seems to represent a risk factor for the development of RHD. In addition, the highly significant frequencies of B allele in both controls and patients without RHD when compared to patients with RHD may indicate that the presence of B allele would be a protective factor against rheumatic carditis. In addition, this polymorphism could be suggested in the differentiation between RF patients with and without RHD.

## Abbreviations

AR: Aortic regurgitation
ASOT: anti-streptolysine O titer
C3: complement 3
C4: complement 4
CRP: C-reactive protein
ESR: erythrocyte sedimentation rate
HF: heart failure
MASPs: MBL-associated serine proteases
MBL: mannose-binding lectin
RF: rheumatic fever
RHD: rheumatic heart disease
SNP: Single nucleotide polymorphism
